# Gaps and opportunities for cervical cancer prevention, diagnosis, treatment and care: evidence from midterm review of the Zimbabwe cervical Cancer prevention and control strategy (2016–2020)

**DOI:** 10.1186/s12889-021-11532-y

**Published:** 2021-07-28

**Authors:** Oscar Tapera, Anna M. Nyakabau, Ndabaningi Simango, Bothwell T. Guzha, Shamiso Jombo-Nyakuwa, Eunice Takawira, Angeline Mapanga, Davidzoyashe Makosa, Bernard Madzima

**Affiliations:** 1 University of Pretoria, School of Health Systems and Public Health, Pretoria, South Africa; 2 Sadtap Health Research Institute (SHRI), Harare, Zimbabwe; 3 Radiotherapy Centre, Parirenyatwa Group of Hospitals, Harare, Zimbabwe; 4 Cancerserve Trust, Harare, Zimbabwe; 5 Department of Obstetrics and Gynaecology, Parirenyatwa Group of Hospitals, Harare, Zimbabwe; 6 College of Health Sciences, University of Zimbabwe, Harare, Zimbabwe; 7 Organization for Public Health Interventions and Development (OPHID), Harare, Zimbabwe; 8 Ministry of Health and Child Care, Directorate of Family Health, Harare, Zimbabwe

**Keywords:** Cervical cancer, Midterm review, Zimbabwe, Strategy, Prevention, Diagnosis, Treatment and care, Mixed methods

## Abstract

**Background:**

Cervical cancer is the fourth most common cancer amongst women globally and it accounts for the majority of cancer deaths among females in Zimbabwe. The objective of this midterm review analysis was to identify the gaps and opportunities for cervical cancer prevention, diagnosis, treatment, and care to inform the next cervical cancer strategy in Zimbabwe.

**Methods:**

A mixed methods approach was used for the midterm review. Secondary data was collected from programme documents, published and grey literature. Primary data was collected in six provinces through key informant interviews with officials and focus group discussions with beneficiaries. After data analysis, a draft report was presented to a technical working group to validate the findings and to fill in any gaps.

**Results:**

This midterm review revealed a myriad of gaps of the strategy particularly in diagnosis, treatment and care of cervical cancer and the primary focus was on secondary prevention. There was no data to quantify the level of awareness and advocacy for cervical cancer prevention. Our results revealed that there was no data on the proportion of women who ever tested for cervical cancer which existed nationally. Our findings suggest that some health facilities were screening women above 50 years old using VIAC, which is an inappropriate approach for those women. Quality control of VIAC and treatment of precancers were not part of the strategy. Pathological services were not efficient and effective due to lack of resources and additionally data on investigations were not routinely collected and available at the national level. Other gaps identified were limited funding, human resources, equipment, and commodities as well as lack of leadership at the national level to coordinate the various components of the cervical cancer programme. There are also numerous opportunities identified to build upon some successes realized to date.

**Conclusions:**

Our findings emphasized the importance of effective and holistic planning in cervical cancer screening programmes in low-resource settings. In addition, huge investments are required in cervical cancer programmes and governments need to take centre role in mobilizing the requisite resources.

## Background

Globally, cervical cancer is the fourth most common cancer amongst women with an estimate of 570,000 new cases and 311,000 deaths in 2018 [1]. At least 90% of the deaths occurred in low- and middle-income countries (LMICs) highlighting the existing global health disparities. Within countries women from the poorest backgrounds, those with lesser education levels, those in rural areas and those facing adverse gender norms are less likely to benefit from timely prevention and detection. They are also more likely to die from cervical cancer than those who come from more socio-economically advantaged groups [2].

Cervical cancer and HIV are interrelated and women living with HIV are four to 10 times more likely to develop cervical cancer and more likely to develop it at a younger age [1, 2]. Despite its potential preventability and curability, cervical cancer remains the most common cancer and is responsible for most cancer deaths among women in Zimbabwe. Out of a total of 7265 new cases, 18% were cervical cancer cases [3]. Vaccination against the human papilloma virus (HPV) infection, screening and treatment of pre-cancerous lesions, early detection and prompt treatment of invasive cancers, and palliative care are all proven, cost-effective strategies that address cervical cancer across the care continuum [4]. Addressing cervical cancer comprehensively, efficiently and effectively at all levels of health care using a multi-sectoral integrated approach will combat the scourge of cervical cancer in Zimbabwe.

The Zimbabwe Cervical Cancer Prevention and Control Strategy (ZCCPCS) is a framework for comprehensive cervical cancer prevention, treatment and control. It is the MoHCC’s commitment to Zimbabwean women and to fulfilling United Nations (UN)‘s sustainable development goals on non-communicable diseases (NCDs). The strategy was premised on scaling up cervical cancer screening using the visual inspection with acetic acid cervicography (VIAC) approach and treatment of precancers using cryotherapy and loop electro-excision procedure (LEEP) across the country [5]. According to World Health Organization (WHO) there are four basic components of cervical cancer control, which encompass: primary prevention, early detection through increased awareness and organized cervical cancer screening programme, diagnosis, treatment and palliative care [6].

Following successful HPV vaccination demonstration projects in two districts Marondera and Beitbridge in 2014 and 2015, Zimbabwe fulfilled the requirements for GAVI financial support of HPV vaccination. Zimbabwe selected school-based vaccination as the primary delivery strategy, with secondary delivery strategies at health facilities and outreach points (such as farms and missions) to reach girls not attending school or who were absent during school vaccinations. Zimbabwe applied for bivalent HPV vaccine which is currently being used with support from UNICEF. HPV vaccination roll out took place in May 2018 and 2019. Advocacy and social mobilization started a month before the vaccination. During the week of vaccination there was a command center in Harare for coordination of mobilization, vaccination and data collection [7].

The objective of this midterm review analysis was to identify the gaps and opportunities for cervical cancer prevention, diagnosis, treatment, and care to inform the next cervical cancer strategy in Zimbabwe.

## Methods

### Design

The midterm review (MTR) adopted a mixed-methods approach analyzing both relevant and available quantitative and qualitative data. Desk review and analysis were carried out using existing secondary data and documentation (e.g. relevant findings from partner needs assessments; monitoring indicators and reports; funding information; human resources and supply data. The majority of the respondents selected were policy makers involved in cervical cancer programmes and were directly or indirectly involved in the development of the existing strategy (see Table 1). In the selection of the respondents at all levels, experience and involvement in any cervical cancer thematic area were considered important criteria. Focus group discussions were also conducted with some purposively selected community members in both urban and rural settings. In addition, these discussions also provided a platform to understand some of key gaps in services that women experience in accessing screening, diagnosis and treatment services.

**Table 1:** Key informants interviewed and their characteristics in terms of areas of responsibility in the strategy implementation

Profession	Number interviewed	Characteristics
**Doctors**	**34**	Policy makers/Implementers
• National (departments at HQ)	9	Policy makers
• Academia	2	Policy makers
• Provincial	13	Implementers
• City Health	2	Policy makers/Implementers
• Private	1	Implementers
• Partners	7	Implementers
Nurses	14 (from 9 institutions)	Implementers
Pharmacist	1	Implementers
M&E Manager	1	Implementers
Health Promotion	1	Implementers
Cytotechnician	1	Implementers
Accountant	1	Implementers
ZNCR Registrar	1	Implementers

### Sample

Six provinces were selected purposively to ensure diverse characteristics relevant to cervical cancer interventions in the country. These provinces were: Harare, Bulawayo, Masvingo, Manicaland, Matabeleland South, and Mashonaland Central (see Table 2). Key informant interviews were conducted with 53 participants, purposively sampled from different relevant departments in the MoHCC at national and provincial levels; representatives from multilateral organizations, private sector and those from NGOs involved in cervical cancer prevention, diagnosis, treatment, palliative care and academia (see Table 1). In addition, focus group discussions (FGDs) were facilitated in the Manicaland and Matabeleland South provinces with beneficiaries of cervical cancer interventions (see Table 3).

**Table 2:** List of provinces selected for Midterm Review in relation to different cervical cancer programme implementation

Province selected	Selection criteria/rationale
Harare	Represents northern region and existence of comprehensive cervical cancer service
Bulawayo	Represents southern region and existence of comprehensive cervical cancer service
Manicaland	Low precancer treatment rates
Masvingo	High precancer treatment rates
Matebeleland South	Pilot province for HPV vaccination
Mashonaland Central	Karanda Mission Hospital provides surgical treatment for HSIL and invasive cancer to women across the country

**Table 3:** Focus groups discussions conducted and characteristics of participants

Province	Type of group	Composition of group
Manicaland	Women who tested positive for VIAC and those with invasive cervical cancer	• 7 women – 3 from Mutare urban and 4 from surrounding rural areas • Mix of ages among the women (30–49 years)
Matabeleland South	Healthy women	• 6 healthy women, 3 from urban and 3 from rural areas, age range 17-49 years

### Data collection

#### Desk review phase

With reference to the baseline findings in the strategy, review documents i.e. the national health strategy, programme documents, publications, UN/WHO guidelines, grey literature were reviewed. These provided theoretical framework for the MTR. Secondary data analysis of reports, presentations and meeting minutes on specifically the cervical cancer strategy were reviewed. Reports, PowerPoint presentations and minutes of different relevant meetings such as Multisectoral Committee, which provided first assessment of progress towards fulfilling the objectives of the ZCCPC strategy were also reviewed. Information/ data included in these documents helped in identifying gaps that were filled through primary data collection.

#### Primary data collection phase

Key informant interviews with representatives from the relevant departments of the MoHCC, NGOs, UN agencies, private sector and academia were conducted. This allowed for probing the high-level sentiments and recommendations on implementing of activities in the ZCCPC strategy. FGDs were facilitated with different groups of women in rural and urban areas in Manicaland and Matabeleland South provinces in Zimbabwe. These provided first-hand information on programme efficiency and effectiveness from the clients’ perspective. Health facilities at different levels from tertiary to primary care were observed in the visited provinces. The scope of the observations included: client flow, the procedure room- space and setup, availability of running water, disposals, availability of IEC materials, equipment availability and functionality, availability and source of medications and skilled human resource availability.

### Data analysis

**Fig. 1:**
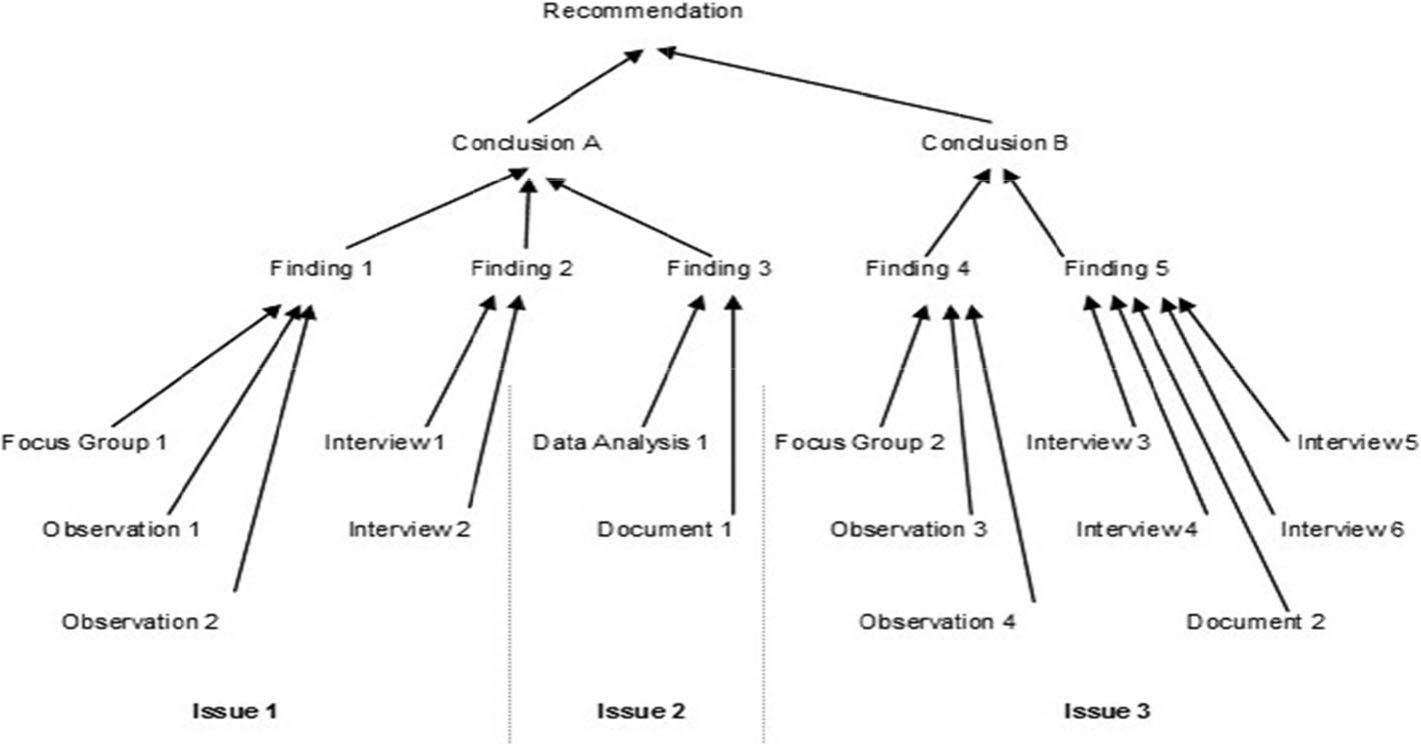
Data Analysis Flow diagram

The overarching questions provided the analytic framework for this midterm review. Thematic analysis was conducted from the key informant interviews [8] and FGDs to generate evidence for this review. Key findings from document research and data collected during field visits were organized based on the objectives of the strategy. To ensure data integrity and factual accuracy throughout the evaluation process, the team engaged in rigorous processes throughout the MTR which allowed for adequate analysis and triangulation as illustrated in Fig. 1.

A synthesis and validation workshop was conducted with stakeholders following the analysis phase to discuss the emerging findings. The workshop provided an opportunity to fill in gaps and harmonise potentially diverging views regarding interpretation of findings. Recommendations were developed in consultation with the stakeholders to ensure they were useful and sustainable.

## Results

Results have been organized based on the main thematic areas of cervical cancer prevention and control strategies as guided by WHO [6] and key pillars of the health system [9].

### Prevention

#### Primary prevention

Most respondents indicated that mass media campaigns, billboards, pamphlets and flyers strengthened and increased awareness and advocacy. Most respondents believed awareness raising was one of the successes of this strategy as evidenced by increasing demand for services shown at all the visited health facilities. However, there was no data to quantify the level of awareness and advocacy for cervical cancer prevention which will be measured during the next ZDHS in 2020. To date, almost a million girls have been vaccinated and the performance of this program has been good, with 86% of the targeted group having been reached.

#### Secondary prevention

There was no data on the proportion of women who ever tested for cervical cancer nationally and this data is expected from the ZDHS scheduled in 2020/1. Some urban health facilities showed that significant numbers of women above 50 years were screened inappropriately with VIAC as shown in Tables 4 and 5. Data from secondary analysis showed that in 2018, 66% of eligible women with precancerous lesions were treated using cryotherapy or loop electrosurgical excision procedure (LEEP) in the country.

**Table 4:** Total Number of women screened in the last 6 months at a Primary Health Facility

Age (years)	< 25	25–49	> 50
HIV +	58	856	121
HIV -	112	474	99
HIV status unknown	0	0	0
**Total**	**170**	**1330**	**220**

**Table 5:** Total Number of women screened in the last 3 months (July–August 2019) at a Tertiary Health Facility

Age (years)	< 25	25–49	> 50
HIV +	18	383	169
HIV -	79	500	187
HIV status unknown	1	6	11
Total	98	889	367

Some of the gaps identified in screening and treatment of precancers were:

- VIAC is less precise compared to other newer technologies on the market like HPV molecular testing which may also be used to screen women above 50 years as well as women with a squamocolumnar junction that is not visible
- VIAC is a manpower intensive screening method. With the high burden of HIV disease and the recommendation of annual cervical cancer screening intervals, this places a strain on the programme and might result in high default rates from follow-up
- VIAC needs to be reviewed especially with a lot of the women now on antiretroviral therapy (ART) requiring annual screening. Other newer screening technologies like HPV molecular testing allow increasing the interval of screening in HIV positive women
- Occasional failure to provide services due to unavailability of sterile packs demotivated clients
- Quality control of VIAC and treatment of precancers were not part of the strategy and the focus has been on numbers of women screened or treated yet quality of the interventions is equally important
- Over diagnosis and overtreatment of low-grade lesions which have adverse effects

### Diagnosis

#### Histological services

Histopathological services are very essential in cervical cancer diagnosis and management though there is not much information about them in the current strategy. In the current programme histopathological services have not been efficient and effective for various reasons. Data on histological investigations which are the definitive diagnostic tests to confirm cervical cancer are not collected and available at a national level. However, data from a tertiary institution in Bulawayo showed that for women who had LEEP in 2016, 2017 and 2018; 62, 77 and 71% of them had histological investigations respectively. In one major tertiary health facility in Bulawayo about 70% of women who had histology results in 2016, 2017 and 2018 had low-grade squamous intraepithelial lesion (LSIL) or normal histology, showing over treatment in health facilities. Over-treatment of pre-cancers has been attributed to the use of VIAC approach which has both low specificity and sensitivity. In addition, the adoption of WHO guidelines which recommended VIAC as a primary screening approach and treating all eligible lesions based on VIAC results could account for the over-treatment observed in our context.

#### Radiological services

Radiological services are very essential and relevant in cervical cancer staging and management; there is not much information about them in the current strategy. Staging and planning of appropriate treatment for women with cervical cancer needs radiological imaging. Before 2018, the following radiological imaging was required to clinically stage patients; chest-x-ray (CXR) and ultrasound scan (USS). These tests are available in most Central Hospitals where the disease is treated. However, there is enough evidence in literature to show that clinical staging is less accurate than radiological staging. The current strategy alludes to setting up of two centers of excellence at United Bulawayo Hospitals (UBH) and Parirenyatwa Group of Hospitals (PGH) to offer comprehensive treatment for women with cervical cancer. Although both canters have computerized tomography (CT) scan machines, UBH has no radiologist and PGH has only one Radiologist. Patients have to pay for these scans and for patients where social welfare is responsible for paying their bills, they still have to pay for consumables like contrast which are very expensive. This has limited the efficiency and effectiveness of radiological services in staging patients with cervical cancer. Both centers have no magnetic resonance imaging (MRI) or positron emission tomography (PET) scans.

#### Blood investigations

There are ongoing trainings for laboratory scientists at the central hospitals, though there is limited access to support blood investigations. Some of the basic tests are not available at all the public laboratories all the time.

Some of the voices from the participants of this evaluation were:

One key informant had this to say “*Cervical cancer staging is very expensive, and most patients can not afford it, government has to come up with a mechanism to subsidize these services*”

“*Women suspected of cervical cancer have to wait for months to be told they have cancer as the results take long*” One FGD participant, Beitbridge

### Treatment

Isolated efforts to improve diagnosis, treatment and palliative care took place during the period of implementation of the strategy. Unfortunately, efforts were neither linked nor integrated and hence opportunities of comprehensive cancer care integration so far have been missed.

Surgery for eligible women with cervical cancer is happening at the tertiary institutions. The key informants from the two tertiary hospitals in Bulawayo reported that all their eligible patients had surgery. However, at the two tertiary institutions in Harare, there were challenges of theatre time, theatre equipment and supplies, nursing staffing levels and facilities for anesthetics and post-operative care and as a result surgery was delayed most of the times. The other contributory factor to the delay in having surgery was the cost of surgery because of the huge out-of-pocket payments. There are persistent data collection and transmission challenges in the registries kept at the various tertiary institutions making it difficult to verify the actual number of women who were eligible for surgery versus those who had surgery.

Chemo-radiation is appropriate treatment for all women with invasive cervical cancer and they will benefit from referral for treatment at tertiary level cancer facilities. Currently there are two state radiotherapy centers in Zimbabwe, Parirenyatwa Group of Hospitals in Harare and Mpilo Central Hospital in Bulawayo. In 2017 a new private oncology center was opened in Harare. On average, approximately 1500 cancer patients are treated at Parirenyatwa Radiotherapy Centre per year. The most common cancer treated is cervical cancer contributing about 30% of the caseload.

In Bulawayo, there was no radiotherapy treatment administered since November 2018 because the machines were not working. All the patients who required treatment were sent to Parirenyatwa Radiotherapy and Oncology Centre, a private facility in Harare or to places outside Zimbabwe commonly South Africa and India. Other potentially curable patients only had palliative care because of lack of money to transfer to the referred centers. Currently, the MoHCC is installing uninterrupted power supply (UPS) to minimize the problem of frequent power cuts which is contributing to the breakdown of the machines at Mpilo and Parirenyatwa Hospital. At Parirenyatwa Radiotherapy and Oncology Centre there are also experiences of frequent machine breakdowns which affect service delivery. We also noted that patient navigation system and tracking of women eligible for treatment and palliative care services does not exist. Some of those who end up at the treatment centers may not receive or complete treatment due to equipment breakdown and shortage of medicines. There is limited access to support for chemo-radiation treatment, leading to heavy out of pocket expenses.

### Palliative care

Palliative care is mostly offered by non-governmental organizations namely Hospice Association of Zimbabwe (HOSPAZ) and Island Hospice and Health Care. MoHCC and partners are in the process of integrating palliative care into the public health system, however; these services are still limited.

Some of the gaps in diagnosis, treatment and palliative care reported were:

- Strategy did not touch much about diagnosis, treatment and palliative care which is a missed opportunity to lobby for more resources for these interventions
- Reliance on out of pocket payments for investigations and treatment leads to treatment delays and high defaulter rates
- Frequent breakdown of diagnostic and therapy machines compromises patient care
- Lack of sustainable accommodation facilities at Parirenyatwa hospital for patients from out of Harare
- Information on cancer treatment outcomes is not readily accessible
- Follow-up of patients throughout the cervical cancer prevention and control continuum remain a challenge
- Statutory instrument 150 authorizes nurses to prescribe morphine but this has not been operationalized
- Health care workers have limited knowledge on cancer and palliative care

Some of the voices from the participants of this evaluation were:

One health worker said “*There is less government commitment to scale-up cancer treatment, and this is seen by the lack of support that we experience in treatment facilities*”

“*Most of the times you are told that radiotherapy machines at Parirenyatwa are not working and one would have travelled long distances to come for that treatment, do you think other people come back for such poor service*?” One FGD participant, Mutare

### Surveillance, monitoring and evaluation

**Fig. 2:**
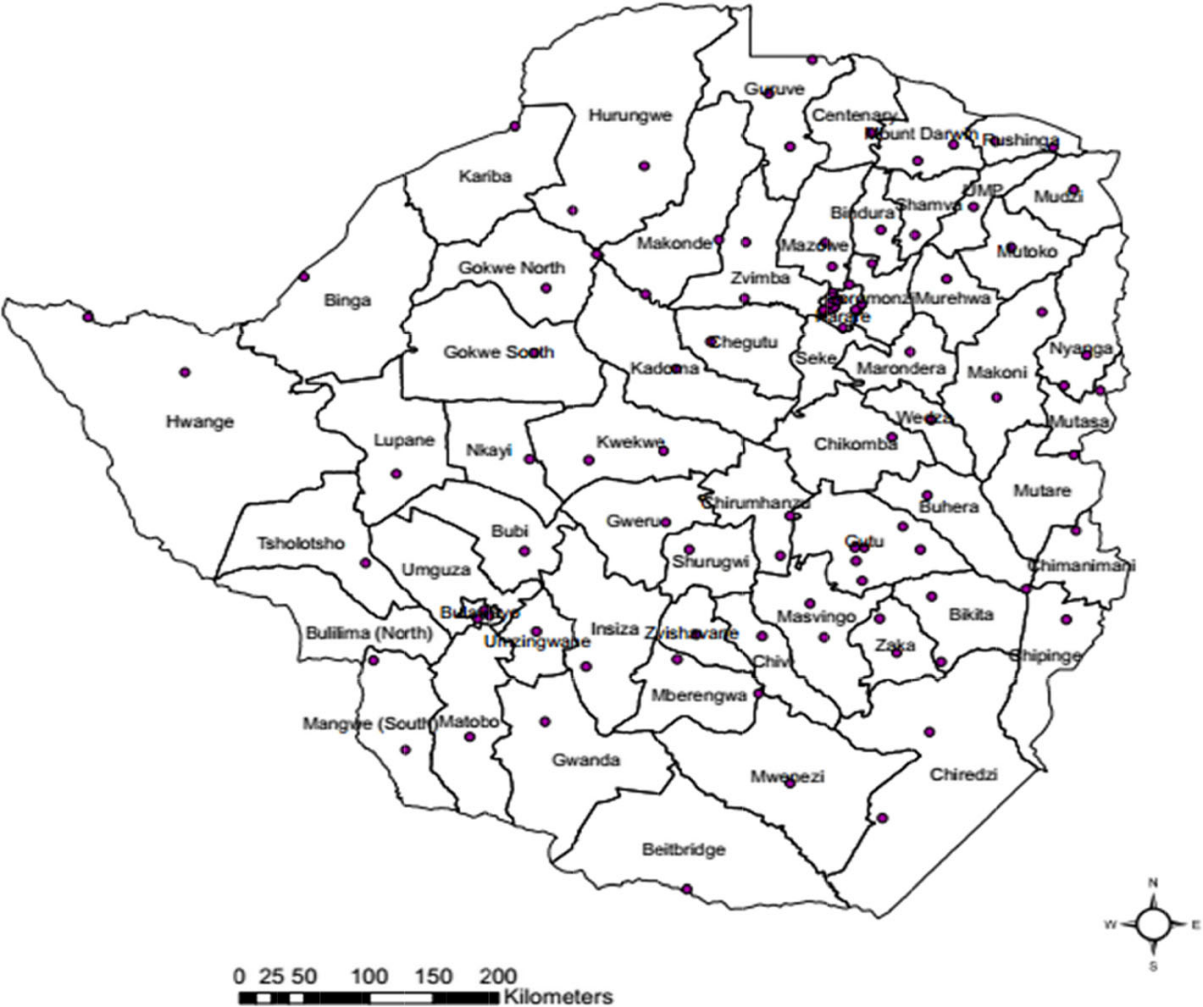
Distribution of public health VIAC sites in Zimbabwe, 2018. (Source – MOHCC, 2017)

Monitoring and evaluation of the strategy did not have a clear framework to define indicators, data sources, collection and reporting for all cervical cancer interventions. Currently 75–80% of the 106 VIAC sites in Zimbabwe (see Fig. 2) are consistently reporting and those not reporting are challenged by limited M&E capacity. Some of the indicators in the strategy are not SMART and therefore difficult to obtain data for. Other indicators in the strategy require national survey data and other data that is not being collected routinely (see Table 6). While the VIAC programme has been noted to have paper-based registers and monthly reporting templates, these tools have some limitations. VIAC indicators have not yet been integrated on the routine MoHCC data collection forms and in the district health information system (DHIS2). There was no evidence of regular data validation, quality control, analysis and dissemination of reports at district and provincial levels. There is limited surveillance of cervical cancer as the National Cancer Registry is mostly active in Harare, Chitungwiza and Bulawayo, although they have started expanding to other provinces.

**Table 6:** Key indicators and targets for the strategy

Key indicators	Baseline (2016) [5]	Midterm results (2019) [5]	Targets (2020)	Comments/Analysis
Reduction in cervical cancer age-specific mortality rate	35.3 per 100,000	46 per 100,000*	33 per 100,000	Mortality may not decrease initially as reporting gets better
Reduction in cervical cancer age-specific incidence rate	56.4 per 100,000	86.1 per 100,000	52 per 100,000	Incidence may not decrease initially as reporting gets better due to screening and better surveillance
Increase in % of women and girls who ever heard about cervical cancer	76%	No data	90%	Data can only be obtained through a national survey such as ZDHS
Increase in % of girls aged 11 years who are vaccinated against HPV	–	86%	80%	Overachievement in this indicator is a good sign of commitment towards primary prevention
Increase in % of women who ever screened for cervical cancer	13%	No data (national survey)	50%	Data can only be obtained through a national survey such as ZDHS
Increase in % of women with precancerous lesions who received treatment.	53%	66%	80%	Significant improvement from the baseline, target realistic.
Increase in % of women eligible for LEEP or suspected with cervical cancer who access histological investigations	–	No data	50%	Data collection needs strengthening for this indicator which can feasibly be collected with the right tools
Increase in % of women with operable cervical cancer who had surgery	–	No data	10%	Data collection needs strengthening for this indicator which can feasibly be collected with the right tools
Increase in % of women with cervical cancer who received radiotherapy and chemotherapy services	–	No data	65%	Data collection needs strengthening for this indicator which can feasibly be collected with the right tools

### Research

Operational research is relevant and should be ongoing in order to have an evidence-based programme. Although the strategy outlined important areas to be targeted by operational research, there is no evidence of comprehensive needs assessment covering the whole continuum of care conducted prior to implementing the strategy. Currently, there is no evidence of cervical cancer operational research studies going on in the country as outlined in the strategy. The current strategy has no operational plan that would have guided the implementation of the research agenda.

### Funding

Financing is key to the success of any programme, however, this strategy was not costed to determine capacity and requirements to meet the intended objectives. The contribution of government to this programme is not clear and most of the resources in screening are funded by NGO partners. Financing of diagnosis, treatment and palliative care is mostly out-of-pocket payments which have hindered access to care. In Zimbabwe, the coverage of health insurance is low and this reduces access to diagnosis, treatment and care among women with cervical cancer.

### Human resources

The current programme is biased towards human resources needed for VIAC screening. Training of nurses in VIAC has been achieved in some facilities to the extent that there is always someone trained in most health facilities. This achievement has been reached through Training of Trainers and on-job trainings. For diagnosis, treatment and palliative care there is a severe shortage of specialists at various levels among them pathologists, oncological surgeons, pediatric oncologists, cytopathologists, counsellors, oncology pharmacists, oncology nurses, physicists and social workers. To address some of these shortages, there are ongoing training programmes for oncologists, radiographers, oncology and palliative care nurses training since 2014. Medical physics training is currently being conducted at National University of Science and Technology (NUST) and University of Zimbabwe. Harare Institute of Technology introduced training of dosimetrist cadres involved in radiotherapy planning and quality assurance of radiotherapy process. International Atomic Energy Agency (IAEA) has been supporting the provision of services, equipment upgrading and training of health personnel at the two cancer treatment centers. A table indicating the gaps of highly specialized manpower involved in cervical cancer diagnosis and treatment is presented in Table 7. The few specialists working in the public sector are poorly motivated and attrition rates of newly qualified staff are high.

**Table 7:** Number of key health personnel, ideal numbers and gaps

Specialties	Currently available	Ideal number	Gaps#	%
Gynaecological oncologists	2	10	8	400%
Gynaecologists	108	128	20	19%
Oncologists	15	64	49	327%
Pathologists	8	64	56	700%
Nuclear Medicine Physician	1	6	5	500%
Radiologist	17	64	47	276%
Oncologist nurses	47	128	115	245%
Medical Physicists	10	20	10	100%
Palliative care specialists	6	64	58	967%

### Materials and commodities

Supply of commodities and equipment to set up VIAC sites was relevant and effective at the onset of the programme. For diagnosis, treatment and palliative care there was no evidence of commodity and equipment supply system in the public sector between 2016 and 2019. The majority of women eligible for diagnosis, chemotherapy and palliative care services rely on private sector players.

#### Equipment

While Mpilo and Parirenyatwa Hospitals have radiotherapy centers, these have been operating sub-optimally due to frequent machine breakdowns, inefficiencies in installations, servicing of equipment and limited human resources to provide the services. Furthermore, the supply chain system and equipment servicing/replacement was not clear in the strategy. Procurement and repair/servicing of equipment is largely centralized and is not clear whose responsibility this falls under in the MoHCC.

#### Commodities

Commodity supply chain is inefficient for VIAC commodities and is not integrated with other health commodities which are distributed quarterly from National Pharmaceutical Company (NatPharm). There was no evidence of stock management system at the health facility level and this was revealed by frequent stock outs of essential commodities such as acetic acid, camera batteries and cryoprobes in some facilities. There was also lack of clarity on commodity back-up system in the event of stock outs as VIAC managers were not clear whose responsibility this was, whether government, non-governmental organization (NGO) partners or health facilities. Currently commodity and equipment procurement and servicing systems are not sustainable in delivering the optimal services for the women population in Zimbabwe.

### Leadership and coordination

Coordination of cervical cancer interventions was identified as one of the major gaps and this has negatively impacted the implementation of programme. While there are numerous partners in the cervical cancer spaces of Zimbabwe, there is evidence of limited coordination among these partners as well as among MoHCC departments. The implementation of the strategy was also not systematic and there was inadequate sensitization of provinces and other stakeholders which is crucial for sustainability. Limited human resources at the central level also compromised implementation as partners and provinces were left to their own discretion in implementing interventions and some of the activities may not have been aligned to the strategy.

## Discussion

The MTR of the Zimbabwe Cervical Cancer Prevention and Control Strategy (2016–2020) revealed that the opinion of most stakeholders was that the strategy led to the raising of awareness especially around cervical cancer screening and this created demand for services. The coverage for the first dose of HPV vaccination achieved by 2019 was 86% against a target of 80% [5] and this was estimated using programme data collected by MoHCC. The HPV vaccination campaign was rolled out in schools in 2018 and 2019 and the target group was girls aged 10–14 years [7]. There was better coverage of cervical cancer screening services using VIAC. Every district in Zimbabwe has a screening site and a total of 106 clinics across the whole country are offering services. Almost two thirds (66%) of women with precancerous lesions received treatment in 2018 against a target of 80%. Tertiary hospitals are offering surgery to eligible women with cervical cancer and there are ongoing efforts towards establishing two centres of excellence in the country. There are opportunities for collaborative, coordinated comprehensive cervical cancer care and research at institutions (public and academic) that can be strengthened and better coordinated by MoHCC. Successes scored in fundraising for ZCCPCS strategy thematic areas including awareness, cervical cancer screening and HPV vaccination set the pace for comprehensive inclusion of diagnosis, treatment and palliative care. There remain some major gaps in the screening, precancer treatment, diagnosis, treatment and palliative care. Some of these gaps emanate from limited resources: funding, human resources, technologies, materials and commodities.

### Prevention

High coverage of HPV vaccination was achieved due to government commitment, good coordination and social mobilization, however, the impact of this interventions in reducing the incidence of cervical precancerous lesions can only be ascertained when the vaccinated girls reach the age of screening (> 25 years based on the current guidance). In screening (VIAC) programme, national data is disaggregated by age into < 16 years, 16–24 years & > 25 years. As a result, it was not possible to measure uptake of screening services for the group 15–49 years using routine programme data and only the ZDHS 2020 will provide national level data. However, a study in Harare by Tapera et al. [10] showed that the proportion of women (at least 25 years old) who were ever screened for cervical cancer was 29% while ZDHS (2015) [11] had reported 24%, showing no significant change. The 50% target by 2020 set in the strategy may not be realistic. In addition, women younger than 25 years and HIV negative and those older than 49 years were also screened, resulting in inefficient use of resources. Young women tend to have HPV infections which are transient and do not require treatment due to possible harms in later pregnancies [12].

There is great potential for the HPV vaccination to continue with good results even to achieve the 90% target set for elimination by WHO [13] should political will persists. There are also opportunities to leverage on the HIV testing and treatment programme to scale-up integration with cervical cancer screening across the country. The synergies could help to improving efficiency, effectiveness and impact of both HIV and cervical cancer interventions. While the Zimbabwe has been doing well in rolling out HPV vaccination, there is still a long way to implementing cervical cancer elimination strategies as recommended by WHO [13]. The country would need to move towards HPV screening and increasing the coverage of pre-cancer treatment services across the country. This will require concerted efforts to mobilize the requisite resources and women/girls to utilize the services. In addition, the impact of the COVID-19 pandemic may crowd out resources and shift priorities in the MoHCC and among its partners [14], hence the elimination agenda may still be far off for Zimbabwe. There are also opportunities for development of a comprehensive and costed strategy for 2021–2024 aligned to the WHO elimination recommendations.

The current cervical cancer screening policy in Zimbabwe indicates that for sexually active women who are HIV negative screening should be done for those between 30 and 49 years using the VIAC approach and if they are VIAC negative they should rescreen every 3 years. For HIV positive women, screening is recommended from 20 to 49 years and rescreening every year using the VIAC approach. Women older than 49 years are not recommended to be screened using VIAC as this may result in false negatives due to lack of the squamocolumnar junction, because of menopause, where pathological transformations may be observed. These women are recommended to be screened with HPV tests or colposcopy. Women are encouraged to screen in health facilities or mobile clinics through community mobilization campaigns and health education sessions offered by health workers. However, Zimbabwe integrated cervical cancer screening with HIV testing and treatment and most NGO partners of the MoHCC have screening programmes biased towards HIV-positive women [5, 6].

While VIAC programme was a commendable approach for screening and early detection of cervical cancer, the precision of the method is quite low and could result in over treatment which may lead to obstetric complications over time [12]. The overall impact on the early detection and identification of pre-cancers and invasive disease may be lower than the strategy was aiming for [12, 13]. In addition, there are some existing technologies and human capacities that are not being fully utilized in the country due to bureaucratic processes and limited knowledge of existing capacities. Currently, “see and treat” approach is challenged during outreach programmes due to heaviness of nitrous oxide tanks. In addition, nitrous oxide is expensive and this has negative implications on the frequency of outreaches which are critical in reaching hard-to-reach populations. Reliance on paper-based registers has limitations of reducing over utilization of services and tracking women referred for further investigations or management. In line with WHO guidelines, the nation should consider piloting and adoption of high precision screening tools i.e. HPV DNA testing for women are at least 30 years old and after every 5 years [15]. It would also be imperative for the MoHCC to conduct a comprehensive assessment of the existing technical capacities in cervical cancer care continuum and leveraging on them. Investment in centralized health information and tracking system platform that uses unique identifiers such as national identification numbers for reporting and tracking women accessing screening, diagnostic, treatment and palliative care should also be considered.

### Diagnosis

It is important that all women who have LEEP or a biopsy of lesions suspicious of cancer receive histology results. This allows for all invasive cancers to be diagnosed and allows for audit of the programme using histology results as the gold standard. Unfortunately, data of women who had access to histological services are kept at health facility level. Zimbabwe’s public histopathological services are centralized in Harare and Bulawayo only. Zimbabwe has a total of eight pathologists; two are based in Bulawayo and six in Harare, this number is far too low. This presents a diagnostic challenge in women with invasive cancers from other provinces. Some VIAC centres were doing LEEPs and were not submitting specimens for processing because the patients had financial challenges for out-of-pocket payment for pathology services. This has a potential of having early invasive cancers missed. In some VIAC sites, women eligible for LEEP were not treated because again they could not afford to send specimens to private laboratories for histology. While, some MoHCC partners have come on board with a pathology service coupon system to alleviate this problem, however, the coupons are not available for every woman. Women with cervical cancer who are suspected outside the screening programme have no access to these coupons. The other challenge is that the majority of the LEEP and punch biopsy specimens are being channeled to private laboratories. The few that are channeled to the public sector have a turnaround time of 6 weeks or more. The main reason for this is lack of capacity in the public sector. The current strategy is also lacking any indicators on how the pathological services are performing. There is need to increase capacity in the public sector by training more pathologists. Partners need to be engaged to see how they can increase capacity and sustainability of histopathological services in the public sector. It is also critical to come up with indicators that can be tracked and audited to improve histopathological services.

In 2018, the International Federation of Gynaecology and Obstetrics (FIGO) revised their staging guidelines to include advanced radiological scanning like computerized tomography (CT) with or without positron emission tomography (PET) and magnetic resonance imaging (MRI) in staging patients with cervical cancer [16]. These factors have impacted negatively on cervical cancer staging and treatment. The CT scan machines at treatment centers are frequently broken down; hence they do not offer sustainable services. One of the reasons given for this is the lack of service contracts for these machines and frequent power cuts which disrupt the machines. For services to be sustainable, more radiologists need to be trained and retained in the public sector. The long-term goal will be to capacitate medical schools in Zimbabwe to offer postgraduate training in radiology. In the meantime, the MoHCC can identify and support doctors to train in radiology in regional universities. There is also need to invest in newer technologies like PET scans and at the same time making sure that the current CT scans are serviced regularly to avoid the frequent breakdowns. This leads to heavy out of pocket expenses and delays women to start treatment as they have to seek for these investigations in private laboratories which are expensive. On the other hand lack of access to blood investigations delays or prevents some patients from required surgery or other definitive treatments [17, 18].

### Treatment

Cervical cancer diagnosis, treatment and palliative care services remain mostly centralized. Treatment in Zimbabwe is still mostly guided by International and not local guidelines. There are still very few oncology surgeons, diagnostic radiologists, radiation oncologists and advanced imaging facilities like MRI scans and these are all prerequisites to making treatment centers of excellence. Best international practice is multidisciplinary meetings (MDT) for every cancer patient before treatment [19]. At one of the tertiary institutions in Harare, this is now part of routine care. However, in most of the other centers, multidisciplinary team meetings are not being conducted prior to planning of treatment which might lead to inappropriate treatment. This evaluation raised issues of women getting suboptimal surgery in peripheral hospitals which offer cheaper services. Local Gynecology Cancer Management guidelines are in the final stages of development as part of Oncology in Zimbabwe Cancer management guidelines and there is need to standardize treatment of cervical cancer patients. Cancer survivors can be ambassadors who champion advocacy, patient navigation and linkages to care. For over 10 years cancer patients attending treatments at Parirenyatwa hospital from out of Harare have faced accommodation challenges and there is need for a long-term sustainable solution to these challenges. There is limited access to support for chemo-radiation treatment; leading to heavy out of pocket expenses which have devastating effects on already vulnerable women in Zimbabwe [19]. There is need for service contract for the machines at both centers and machine replacement plan for the aging machines to ensure regular uninterrupted service. The five national treatment machines are all linear accelerators which require resident engineer for frequent servicing. Local training of these engineers should be considered. The MoHCC and partners (including IAEA) should consider installation of an additional radiotherapy machine at the existing centers or at a third center. The MoHCC should embark on strengthening in-service and pre-service training of health workers on cancer and strengthening of multi-disciplinary teams in cancer management to improve diagnosis, treatment and care of cervical cancer patients.

### Palliative care

Palliative care integration has the potential to strengthen comprehensive cancer care. The MoHCC developed and rolled out a palliative care policy in 2014 and a draft strategy exists. There is a statutory instrument SI150 which allows palliative care nurses to prescribe morphine for pain management which needs operationalization. One of the limitations of palliative care is opiophobia which emanates from lack of knowledge and negative perceptions of the approach [20]. Palliative care is not covered in detail in the strategy although advanced cervical cancer burden is high in the country [1, 3]. One recent study by Tapera et al. [21] showed that only 13% of women with advanced cervical cancer had access to palliative care in Harare and this finding supports what was reported in our review. There is need for the government to strengthen the integration of palliative care into the health delivery system in line with international recommendations [19, 21].

### Surveillance, monitoring and evaluation

Our review showed piecemeal surveillance and monitoring and evaluation system for cervical cancer with different NGOs setting up their own systems or frameworks to satisfy their reporting requirements. However, there are significant gaps in the measurement of outputs and outcomes from cervical cancer interventions in the country emanating from having a poor national surveillance system and lack of a monitoring and evaluation framework with measureable indicators. While the MoHCC has started incorporating cervical cancer screening data in T5 and DHIS2 system, this will require good coordination with NGO partners and human resource capacity to optimize. It is imperative that MoHCC develops a monitoring and evaluation framework which will outline clear targets, indicators, data flow and quality issues for comprehensive cervical cancer interventions for the next strategy in line with WHO guidelines [6].

### Funding

The majority of cervical cancer interventions including human resources, materials and commodities are funded by NGO partners, with little or no support from the fiscus. This poses sustainability challenges on cervical cancer programme in the country. While there was some support for screening and treatment of precancers, there was limited support for diagnosis, treatment and care. This predisposes families to out-of-pocket financing given the low coverage of health insurance in Zimbabwe [11, 22]. In addition, the benefits of health insurance have been negatively impacted by the economic challenges further reducing access among those with health insurance. There is need for government financial commitment to the whole cervical cancer prevention and control strategy. Exploration of public private partnerships and mobilization of traditional and non-traditional donors, private sector and NGOs should be considered to help with financing strategy implementation. The MoHCC should consider a costed operational plan for the next strategy and to use a business case approach to mobilize resources.

### Limitations of midterm review

The evaluation had some limitations namely unavailability of some key informants and focus group participants which could potentially bias the findings**.**

## Conclusions

Our findings emphasized the importance of effective and holistic planning in cervical cancer screening programmes in low-resource settings. In addition, huge investments are required in cervical cancer programmes and governments need to take the centre role in mobilizing the requisite resources. Development and implementation of cervical cancer strategies should rely heavily on strong political will and collaboration of governments, private sector and NGO partners, using lessons learnt from previous strategy reviews. This is crucial for more sustainable interventions to reduce the morbidity and mortality of cervical cancer in low-resource settings. It is recommended for limited-resource countries to develop and pilot elimination strategies in one or two subnational geographical locations. The pilots would inform some key lessons learnt and allow Ministries of Health and their partners to plan better to scale-up the strategies. Finally, improvement of communication strategy is imperative to operationalize the cervical cancer elimination strategies as recommended on WHO across nations.

## Data Availability

The data used and/or analyzed during the evaluation are available from the corresponding author on reasonable request.

## Abbreviations

ART: Antiretroviral Therapy
CT: Computerized Tomography
CXR: Chest X-ray
DHIS: District Health Information System
DNA: Deoxyribonucleic Acid
FGD: Focus Group Discussion
FIGO: International Federation of Gynaecology and Obstetrics
HIV: Human Immunodeficiency Syndrome
HPV: Human Papilloma Virus
IAEA: International Atomic Energy Agency
LEEP: Loop Electro-Excision Procedure
LMIC: Low- and Middle-Income Countries
LSIL: Low-grade Squamous Interepithelial Lesion
MDT: Multidisciplinary Teams
MoHCC: Ministry of Health and Child Care
MRI: Magnetic Resonance Imaging
MTR: Midterm Review
NatPharm: National Pharmaceutical Company
NCD: Non-Communicable Diseases
NGO: Non-Governmental Organization
NUST: National University of Science and Technology
PET: Positron Emission Tomography
PGH: Parirenyatwa Group of Hospitals
SMART: Specific, Measurable, Attainable, Realistic and Time-bound
UBH: United Bulwayo Hospitals
UN: United Nations
UPS: Uninterrupted Power Supply
USS: Ultrasound Scan
VIAC: Visual Inspection with Acetic Acid Cervicography
WHO: World Health Organization
ZCCPCS: Zimbabwe Cervical Cancer Prevention and Control Strategy
ZDHS: Zimbabwe Demographic and Health Survey
